# PF-3845, a Fatty Acid Amide Hydrolase Inhibitor, Directly Suppresses Osteoclastogenesis through ERK and NF-κB Pathways In Vitro and Alveolar Bone Loss In Vivo

**DOI:** 10.3390/ijms22041915

**Published:** 2021-02-15

**Authors:** Hye-Jung Ihn, Yi-Seul Kim, Soomin Lim, Jong-Sup Bae, Jae-Chang Jung, Yeo-Hyang Kim, Jin-Woo Park, Zhao Wang, Jeong-Tae Koh, Yong-Chul Bae, Moon-Chang Baek, Eui-Kyun Park

**Affiliations:** 1 Cell and Matrix Research Institute, Kyungpook National University, Daegu 41944, Korea; hjpihn@hanmail.net; 2 Department of Oral Pathology and Regenerative Medicine, School of Dentistry, IHBR, Kyungpook National University, Daegu 41940, Korea; ekdms121212@naver.com (Y.-S.K.); friendship1240@hanmail.net (S.L.); 3 College of Pharmacy, CMRI, Research Institute of Pharmaceutical Sciences, Kyungpook National University, Daegu 41566, Korea; baejs@knu.ac.kr; 4 Department of Biology, College of Natural Sciences, Kyungpook National University, Daegu 41566, Korea; jcjung@knu.ac.kr; 5 Department of Pediatrics, School of Medicine, Kyungpook National University, Daegu 41944, Korea; kimhmd@knu.ac.kr; 6 Department of Periodontology, School of Dentistry, Kyungpook National University, Daegu 41940, Korea; jinwoo@knu.ac.kr; 7 Department of Pharmacology and Dental Therapeutics, School of Dentistry, Chonnam National University, Gwangju 61186, Korea; htwz1996@163.com (Z.W.); jtkoh@chonnam.ac.kr (J.-T.K.); 8 Department of Oral Anatomy and Neurobiology, School of Dentistry, Kyungpook National University, Daegu 41940, Korea; ycbae@knu.ac.kr; 9 Department of Molecular Medicine, CMRI, School of Medicine, Kyungpook National University, Daegu 41944, Korea

**Keywords:** PF-3845, osteoclastogenesis, bone resorption, alveolar bone loss, ERK

## Abstract

Alveolar bone loss, the major feature of periodontitis, results from the activation of osteoclasts, which can consequently cause teeth to become loose and fall out; the development of drugs capable of suppressing excessive osteoclast differentiation and function is beneficial for periodontal disease patients. Given the difficulties associated with drug discovery, drug repurposing is an efficient approach for identifying alternative uses of commercially available compounds. Here, we examined the effects of PF-3845, a selective fatty acid amide hydrolase (FAAH) inhibitor, on receptor activator of nuclear factor kappa B ligand (RANKL)-mediated osteoclastogenesis, its function, and the therapeutic potential for the treatment of alveolar bone destruction in experimental periodontitis. PF-3845 significantly suppressed osteoclast differentiation and decreased the induction of nuclear factor of activated T-cells cytoplasmic 1 (NFATc1) and the expression of osteoclast-specific markers. Actin ring formation and osteoclastic bone resorption were also reduced by PF-3845, and the anti-osteoclastogenic and anti-resorptive activities were mediated by the suppression of phosphorylation of rapidly accelerated fibrosarcoma (RAF), mitogen-activated protein kinase (MEK), extracellular signal-regulated kinase, (ERK) and nuclear factor κB (NF-κB) inhibitor (IκBα). Furthermore, the administration of PF-3845 decreased the number of osteoclasts and the amount of alveolar bone destruction caused by ligature placement in experimental periodontitis in vivo. The present study provides evidence that PF-3845 is able to suppress osteoclastogenesis and prevent alveolar bone loss, and may give new insights into its role as a treatment for osteoclast-related diseases.

## 1. Introduction

Periodontitis, a prevalent inflammatory oral disorder, is the primary cause of tooth loss in adults [1]. The accumulation of microbial plaque induces inflammation, and an excessive host immune response drives the destruction of the tooth-supporting structures, including the periodontal ligament and alveolar bone [2]. During the progression of periodontitis, the secretion of pro-inflammatory cytokines promotes osteoclast formation and osteoclastic bone resorption, leading to alveolar bone destruction and subsequent tooth loss [3]. Furthermore, as there appears to be an association between periodontitis and various systemic conditions, the effective management of causative factors and appropriate treatment methods are especially important.

Multinucleated osteoclasts are mainly responsible for mineralized bone degradation, and are formed by the fusion of mononuclear progenitors originating from stem cells in the bone marrow [4]. Macrophage-colony stimulating factor (M-CSF) and receptor activator of nuclear factor-κB ligand (RANKL) are key cytokines involved in osteoclastogenesis and bone-resorbing function [5]. The binding of RANKL to the cognate receptor RANK of osteoclast precursors activates intracellular signaling cascades, which ultimately results in the activation of the nuclear factor of activated T-cells cytoplasmic 1 (NFATc1), thereby promoting the transcription of target genes related to osteoclast differentiation [6]. Since bone destruction caused by excessive osteoclast formation and subsequent bone resorption is implicated in skeletal diseases, including periodontitis, therapeutic agents possessing anti-osteoclastogenic and anti-resorptive activities can be effective and potentially useful as treatments.

As part of our ongoing research on the identification of potential anti-osteolytic agents by drug repurposing, Food and Drug Administration (FDA)-approved drugs were initially screened for their ability to suppress the differentiation of bone marrow macrophages (BMMs) into osteoclasts. Using a tartrate-resistant acid phosphatase (TRAP) assay, we found that PF-3845 inhibited RANKL-mediated osteoclast formation in vitro. PF-3845, a potent and irreversible inhibitor of fatty acid amide hydrolase (FAAH), has anti-nitrosative and anti-inflammatory activities against acute stress [7]. It was shown to protect against traumatic brain injury (TBI)-induced neuroinflammation and neurodegeneration via the activation of cannabinoid type 1 and 2 receptors in a mouse model [8]. Wasilewski et al. have demonstrated its beneficial properties for reducing the viability, migration, and invasion of colon adenocarcinoma Colo-205 cells [9]. However, there are no reports on the potential effect of PF-3845 on osteoclastogenesis and bone resorption. Here, we investigated the anti-osteoclastogenic effect of PF-3845 and the molecular mechanisms underlying its suppressive action. Furthermore, an experimental periodontitis model in mice was used to assess the in vivo efficacy of PF-3845.

## 2. Results

### 2.1. Effect of PF-3845 on Osteoclast Differentiation

**Figure 1:**
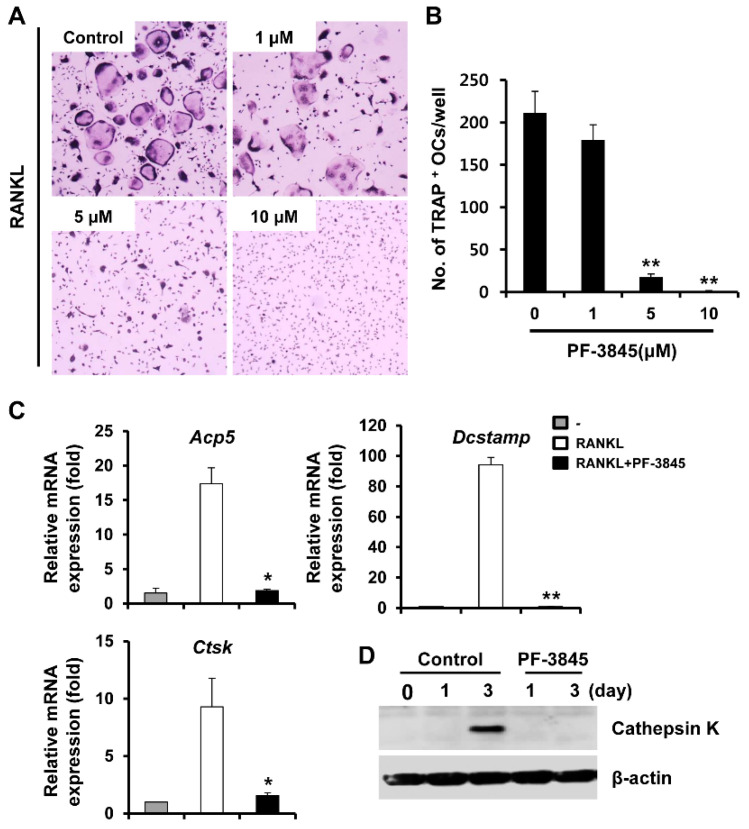
The effect of PF-3845 on receptor activator of nuclear factor kappa B ligand (RANKL)-induced osteoclast (OC) differentiation. (**A**) Bone marrow macrophages (BMMs) were incubated with macrophage-colony stimulating factor (M-CSF) (10 ng/mL) and RANKL (20 ng/mL) in the presence of indicated concentrations of PF-3845. After four days, tartrate-resistant acid phosphatase (TRAP) staining was performed. (**B**) The number of TRAP-positive cells containing three or more nuclei was calculated. (**C**) BMMs were cultured with M-CSF (10 ng/mL) and RANKL (20 ng/mL) in the presence of 10 μM PF-3845 for four days. The mRNA expressions of osteoclast-specific genes were assessed by real-time PCR analysis, tartrate-resistant acid phosphatase (*Acp5*), dendritic cell-specific transmembrane protein (*Dcstamp*), and cathepsin K (*Ctsk*). (**D**) BMMs were cultured in an osteoclastogenic medium with the vehicle or 10 μM PF-3845 for an indicated number of days. Cathepsin K protein expression was evaluated by immunoblotting. * *p* < 0.05, ** *p* < 0.01 (two-tailed Student’s *t*-test).

The effect of PF-3845 on RANKL-induced osteoclastogenesis was investigated using mouse BMMs. BMMs stimulated with M-CSF and RANKL differentiated into TRAP-positive multinucleated cells (MNCs), whereas osteoclast formation was significantly inhibited by PF-3845 in a concentration-dependent manner (Figure 1A). More than 90% inhibition was observed with 5 or 10 μM PF-3845 treatment (Figure 1B), and the anti-osteoclastogenic effect was not attributable to cytotoxicity, as assessed by 3-(4,5-dimethylthiazol-2-yl)-2,5-diphenyltetrazolium bromide (MTT) assay (data not shown).

We then examined the expression levels of genes involved in osteoclast differentiation to confirm the suppressive effect of PF-3845 on osteoclastogenesis. Consistent with the results of TRAP staining, osteoclast marker genes, including *Acp5*, *Dcstamp*, and *Ctsk*, were upregulated in response to RANKL, whereas the expression of these genes was significantly reduced in PF-3845 (10 μM)-treated cells (Figure 1C). In addition, the protein level of cathepsin K, assessed by Western blot, was lower, as compared to that of the vehicle-treated control (Figure 1D).

### 2.2. Effect of PF-3845 on RANKL-Mediated NFATc1 Induction and Actin Ring Formation

**Figure 2:**
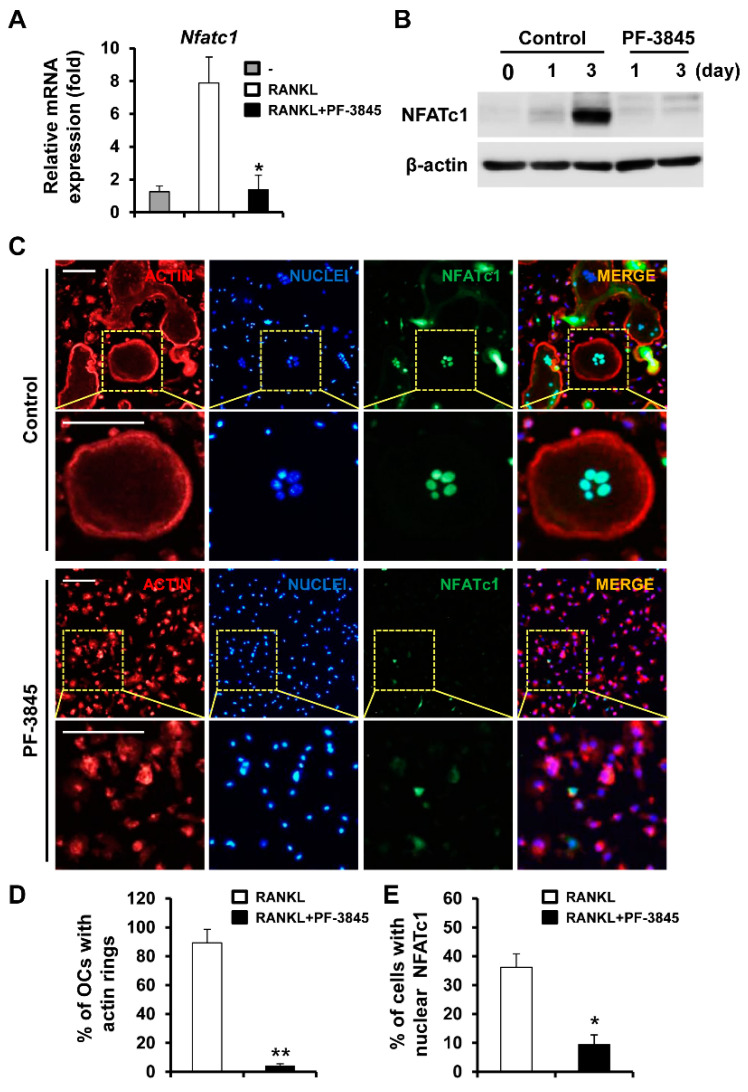
The effect of PF-3845 on nuclear factor of activated T-cells cytoplasmic 1 (NFATc1) expression and actin ring formation. BMMs were cultured in an osteoclastogenic medium with the vehicle or 10 μM PF-3845 for four days (**A**,**C**–**E**) or the number of days indicated (**B**). The mRNA (**A**) and protein (**B**) expression of NFATc1 were examined. (**C**) After four days of culture, the cells were probed with anti-NFATc1 antibody followed by staining with rhodamine-conjugated phalloidin and 4′,6-diamidino-2-phenylindole dihydrochloride (DAPI) to visualize F-actin and nuclei, respectively. The percentage of (**D**) osteoclasts with actin rings and (**E**) nuclear NFATc1-positive cells was assessed. Yellow dashed box: magnified region, scale bar, 50 μm. * *p* < 0.05, ** *p* < 0.01 (two-tailed Student’s *t*-test).

Given that NFATc1 regulates osteoclast-specific genes responsible for the formation and function of osteoclasts [10], we examined the effect of PF-3845 on NFATc1 expression. As expected, the levels of NFATc1 mRNA and protein were lower in PF-3845 (10 μM)-treated cells (Figure 2A,B), which correlated with the reduced expression of its target genes. The immunofluorescence results showed that multinucleated osteoclasts in the control group at day four expressed NFATc1 in the nuclei, while PF-3845 treatment significantly decreased the expression and nuclear localization of NFATc1 (Figure 2C). In the presence of PF-3845, approximately 10% of cells expressed nuclear NFATc1 (Figure 2E).

Actin ring structures in mature osteoclasts are closely associated with the resorption of mineralized matrix. Multinucleated osteoclasts with actin rings were generated in response to RANKL stimulation, while 10 μM PF-3845 treatment strongly reduced actin ring formation (Figure 2C,D).

### 2.3. Effect of PF-3845 on Bone-Resorbing Activity

**Figure 3:**
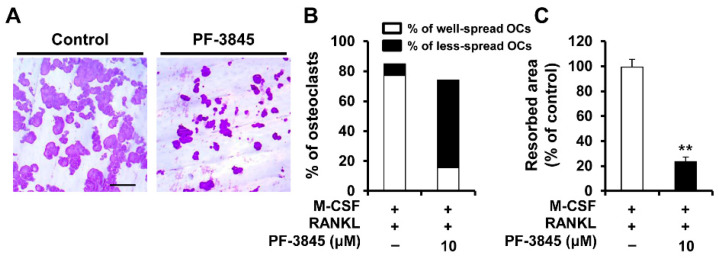
The effect of PF-3845 on the formation of resorption pits. BMMs were grown on bone slices in an osteoclastogenic medium for three days and then the vehicle or 10 μM PF-3845 was added. (**A**) After 24 h, the bone slices were stained with hematoxylin to examine the resorbed areas. Quantification of the percentage of (**B**) well-spread or less-spread osteoclasts and (**C**) resorption area. Scale bar, 100 μm. ** *p* < 0.01 (two-tailed Student’s *t*-test).

Mature osteoclasts degrade mineralized tissue by secreting protons and proteolytic enzymes into the resorption lacuna [11]. To investigate whether PF-3845 affects the bone-resorbing activity of osteoclasts, we performed a pit formation assay and assessed bone resorption activity by measuring the resorbed area. In the vehicle-treated control, numerous large pits were generated on the bone slices (Figure 3). However, treatment with PF-3845 significantly decreased the percentage of the resorbed area compared with that of the control (Figure 3).

### 2.4. Effect of PF-3845 on RANKL-Mediated Signal Transduction

**Figure 4:**
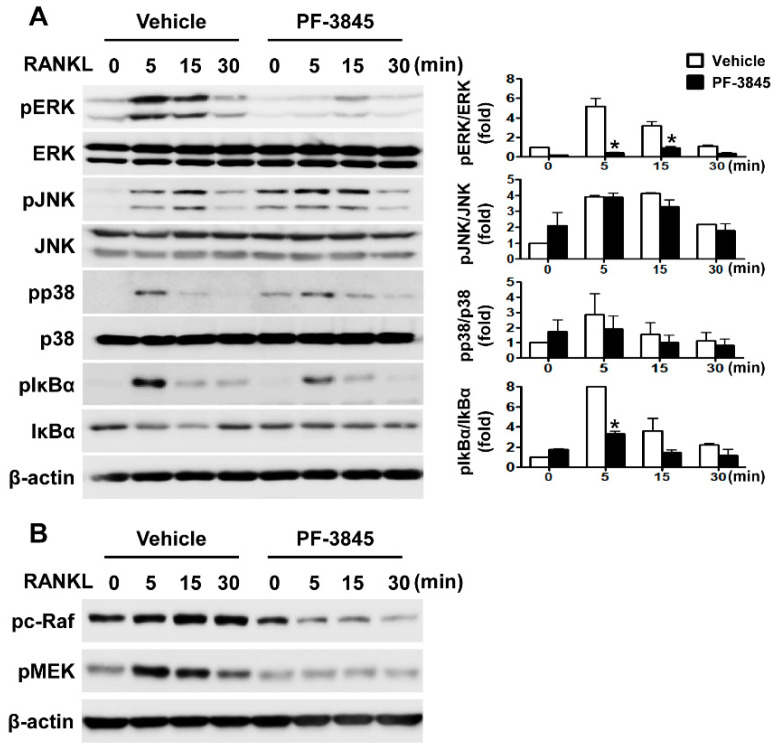
The effect of PF-3845 on RANKL-induced signaling in BMMs. (**A**,**B**) BMMs incubated in a serum-free medium for 5 h were treated with the vehicle or PF-3845 (10 μM). After incubation for 1 h, the cells were treated with RANKL (50 ng/mL) for the indicated time periods. Cell lysates were subjected to immunoblot analysis. (**A**) The expression of phosphorylated-ERK, -JNK, -p38, and -IκBα was assessed. The total ERK, JNK, p38, IκBα, and β-actin were used as the loading controls. (**B**) Phosphorylation of c-RAF and MEK were determined by Western blot analysis. β-actin was used as the loading control. * *p* < 0.05 (two-tailed Student’s *t*-test).

Since RANKL is essential for osteoclast differentiation, we investigated the influence of PF-3845 on intracellular signaling pathways activated by this cytokine in BMMs to elucidate the molecular mechanism of action. RANKL stimulation led to the phosphorylation of MAPKs and nuclear factor κB (NF-κB) inhibitor (IκBα) in the vehicle-treated control, each reaching a peak within 5 to 15 min (Figure 4A). In contrast, phosphorylation of extracellular signal-regulated kinase (ERK) and IκBα was significantly reduced by 10 μM PF-3845 treatment (Figure 4A). As PF-3845 affected the RANKL-mediated activation of ERK, we further investigated its effects on upstream signaling molecules. As shown in Figure 4B, Raf/MEK phosphorylation was strongly suppressed when BMMs were pretreated with PF-3845.

### 2.5. Effect of PF-3845 on Alveolar Bone Loss in Experimental Periodontitis

**Figure 5:**
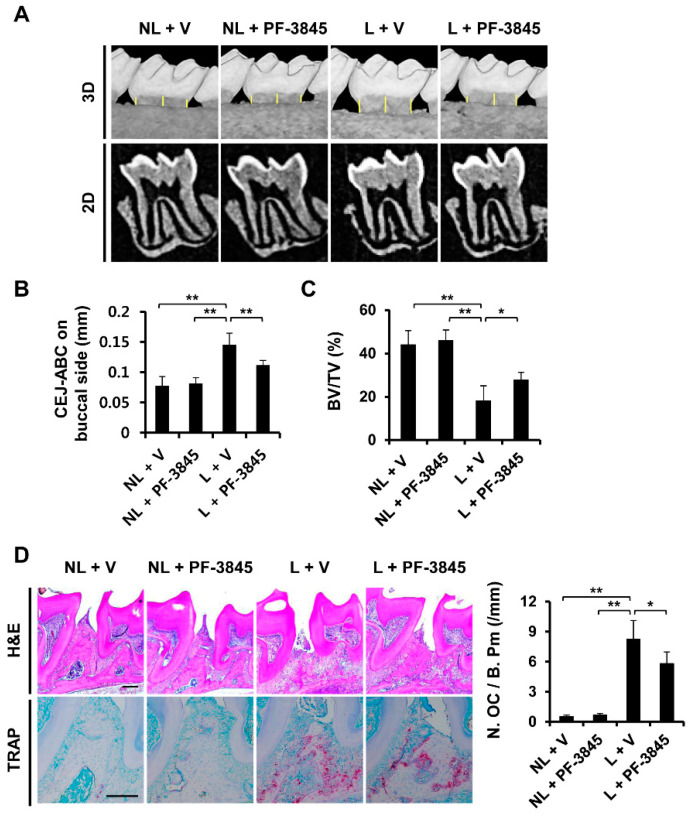
The effect of PF-3845 on alveolar bone loss in mice with experimental periodontitis. (**A**) Three-dimensional and two-dimensional micro-CT images of maxillary second molars in the nonligation with vehicle (NL + V), nonligation with PF-3845 (NL + PF-3845), ligation with vehicle (L + V), and ligation with PF-3845 (L + PF-3845) groups. (**B**) The cementoenamel junction (CEJ)–alveolar bone crest (ABC) distance (indicated by yellow lines) and (**C**) bone volume fraction (BV/TV) were measured. (**D**) The maxillae were decalcified, sectioned, and stained with hematoxylin and eosin (H & E) and TRAP. The number of osteoclasts per bone perimeter [N. OC/B. Pm (/mm)] was measured (right graph). n = 5 in each group. Scale bar, 200 μm. * *p* < 0.05, ** *p* < 0.01 (ANOVA with Tukey’s post hoc).

Given that PF-3845 exhibited anti-osteoclastogenic and anti-resorptive activities in vitro, we next examined whether PF-3845 treatment could prevent alveolar bone loss using a ligature-induced periodontitis model in mice. As observed in our previous studies, severe alveolar bone resorption around the molar occurred in the ligated groups (Figure 5A). The distance between the cementoenamel junction (CEJ) and the alveolar bone crest (ABC) in the ligation with vehicle (L + V) group was increased by 87.53%, as compared to that in the nonligation with vehicle (NL + V) group (Figure 5A). However, the CEJ–ABC distance in mice injected with PF-3845 was 0.11 ± 0.01 mm, which was lower than that of the ligation with vehicle (L + V) group (Figure 5B). In addition, an obvious reduction in bone volume fraction was observed in the ligation with vehicle (L + V) group, but PF-3845 administration attenuated ligature-induced decrease in the ratio of BV/TV (Figure 5C). Hematoxylin and eosin (H&E) staining also showed greater bone loss around the ligated teeth when compared with the nonligation group (Figure 5D). Even though the placement of ligatures caused bone resorption, PF-3845 administration ameliorated such alveolar bone loss (Figure 5D,E,H). In addition, TRAP staining results revealed that ligation accelerated the formation of TRAP-positive osteoclasts. However, mice treated with PF-3845 exhibited a decrease in the number of osteoclasts, as compared with the ligation with vehicle (L + V) group (Figure 5D, TRAP), suggesting that PF-3845 efficiently inhibits osteoclast formation and bone destruction in vivo and may prevent alveolar bone loss.

### 2.6. Effect of Other FAAH Inhibitors on Osteoclast Differentiation

**Figure 6:**
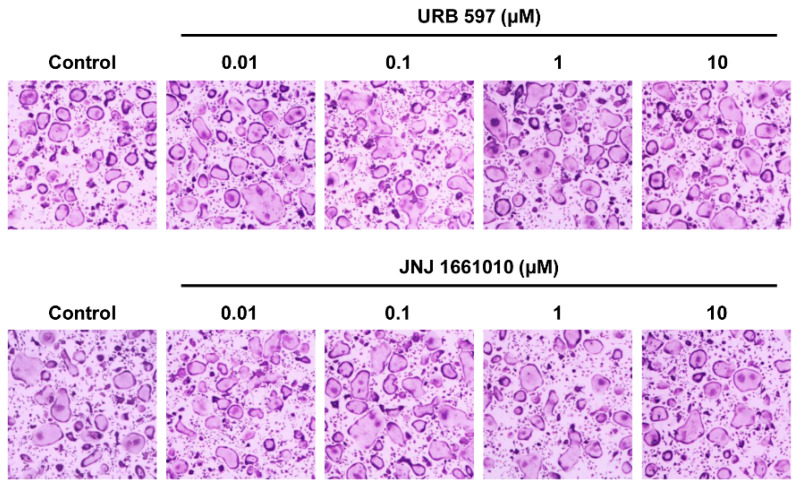
The effect of two other fatty acid amide hydrolase (FAAH) inhibitors, URB597 and JNJ1661010, on osteoclast differentiation. BMMs were cultured in an osteoclastogenic medium with the vehicle or several concentrations of FAAH inhibitors, URB597 (**upper** panel), or JNJ1661010 (**lower** panel). The cells were stained for TRAP.

We next examined the effects of two other FAAH inhibitors, URB597 and JNJ1661010, on RANKL-induced osteoclast differentiation to determine if the suppressive effects of PF-3845 were related to the inhibition of FAAH. Unlike PF-3845, the other inhibitors did not affect osteoclast formation (Figure 6).

## 3. Discussion

Since efficient anti-resorptive therapies for protection against alveolar bone destruction in periodontitis are limited, there is a need for the development of promising candidate drugs. Drug repositioning, a way of identifying novel indications for approved drugs, is considered to be an attractive drug development strategy because of its positive aspects [12]. Here, we report that PF-3845 exhibits anti-osteoclastogenic and anti-resorptive activities. PF-3845 significantly suppressed RANKL-stimulated osteoclast differentiation and reduced the formation of resorption pits in vitro. In addition, it prevented alveolar bone destruction caused by ligature placements in vivo.

RANKL-RANK signaling required for the differentiation of osteoclast precursors into bone-resorbing osteoclasts induces the principal regulator NFATc1, subsequently upregulating the mRNA levels of osteoclast marker genes [6]. Various protein-kinase-mediated signaling pathways are activated by RANK and involved in osteoclastogenesis and activation. The genetic or pharmacological inhibition of ERK impairs osteoclast differentiation and function, providing evidence of the important role of the ERK pathway [13,14]. In addition, the blockade of ERK signaling attenuates inflammatory osteolysis in mice, supporting the consideration of RAF/MEK/ERK signaling as a therapeutic target for osteoclast-related diseases [15]. We observed that PF-3845 attenuated the phosphorylation of RAF/MEK/ERK molecules (Figure 4), indicating that PF-3845 inhibition occurs through suppression of the RAF/MEK/ERK pathway. The present study also revealed that PF-3845 reduced the protein and mRNA levels of NFATc1, as well as those of its target genes, including *Acp5*, *Dcstamp*, and *Ctsk* (Figure 1 and Figure 2). Among them, the central role of DCSTAMP in the fusion of osteoclast precursors during osteoclast differentiation is well established [16,17]. *Dcstamp*-deficient mice exhibited an osteopetrotic phenotype due to the failure of osteoclast multinucleation and reduction in osteoclastic bone resorption [16]. Mature osteoclasts formed by the cellular fusion of osteoclast progenitors adhere tightly to the bone surface via the formation of F-actin rings, which are important for the resorption process [18]. The degradation of the bone matrix by osteoclasts takes place within the sealing zone formed by the actin ring structure [19]. PF-3845 treatment impaired osteoclast multinucleation as well as actin ring formation, thereby suppressing osteoclastic bone resorption (Figure 2D and Figure 3), indicating that PF-3845 may be a potential anti-osteoclastogenic and anti-resorptive agent.

The ligature-induced periodontitis model is useful for studying the mechanism of periodontal disease and examining the potential preventative and/or therapeutic efficacy of agents [20,21,22]. Ligature placement around the second molar accelerates inflammation in the periodontal tissue and induces alveolar bone loss [23], and the results from our previous research are similar to these findings [21,22]. In the present study, severe bone destruction occurred in mice subjected to ligature, corresponding to an increased CEJ–ABC distance (Figure 5A). In accordance with the anti-osteoclastogenic effect in vitro, PF-3845 administration attenuated alveolar bone resorption caused by ligature placement, and the number of osteoclasts was significantly decreased (Figure 5A), indicating the therapeutic potential of PF-3845 as a bone-protective agent in periodontal diseases. Even though PF-3845 exhibited potent inhibitory effects on RANKL-induced osteoclast formation, we observed that PF-3845 suppressed IL-6 mRNA expression, but slightly induced TNF-α mRNA expression in vitro (data not shown), indicating that inflammatory responses might be partially attenuated by PF-3845, which may explain the mild protection in vivo.

PF-3845 is a selective inhibitor of FAAH, and the effects caused by the FAAH blockade are comparable to those generated by cannabinoid (CB) receptor agonists [24]. The CB2-specific agonist HU-308 suppresses the differentiation of RAW 264.7 cells into osteoclasts in response to RANKL in vitro and limits ovariectomy (OVX)-induced bone loss in vivo [25]. Rossi and colleagues also reported the stimulatory effects of a CB2 antagonist on osteoclastogenesis [26]. In agreement with these findings, PF-3845 showed inhibitory activities against osteoclastogenesis and bone resorption and attenuated ligation-induced alveolar bone loss by inhibiting osteoclast formation. Meanwhile, there are contradictory findings regarding the role of the endocannabinoid system in the regulation of osteoclast differentiation and function. Idris et al. reported that an endogenous CB agonist, anandamide, enhances osteoclast formation, and pharmacological antagonists of CB receptors cause osteoclast apoptosis and protect against bone loss in ovariectomized mice [27]. In the present study, we observed that two other FAAH inhibitors, URB597 and JNJ1661010, did not attenuate the differentiation of BMMs into osteoclasts. Although the reasons for the opposing effects are not clear thus far, the possibility of off-target effects cannot be ruled out [28]. In particular, RANKL-induced activation of the RAF/MEK/ERK and NF-κB pathways was specifically inhibited by PF-3845, suggesting that it may also inhibit RAF/IKK or upstream molecule(s) in the RANK signaling pathways.

Although further study is required to determine appropriate dosage and treatment strategies, the present study demonstrates that PF-3845 has anti-osteoclastogenic and anti-resorptive activities via suppression of ERK/NF-κB activation. Additionally, PF-3845 administration prevents alveolar bone loss caused by experimental periodontitis in mice through reduced osteoclast formation, suggesting that PF-3845 may be beneficial for treating osteoclast-related bone diseases.

## 4. Materials and Methods

### 4.1. Mice and Reagents

Six-week-old male mice were purchased from Dae Han Bio Link (Chungbuk, Korea). All animal experiments were approved by the committees on the care and use of animals in research at Kyungpook National University and were conducted in accordance with the guidelines for the care and use of laboratory animals (KNU 2016-0147). PF-3845 and 3-(4,5-dimethylthiazol-2-yl)-2,5-diphenyltetrazolium bromide (MTT) were obtained from Sigma-Aldrich (St. Louis, MO, USA). Fetal bovine serum (FBS) and α-minimal essential medium (α-MEM) were purchased from Gibco BRL (Grand Island, NY, USA). Recombinant M-CSF and RANKL were obtained from R & D Systems (Minneapolis, MN, USA).

### 4.2. Osteoclast Differentiation

BMMs were prepared as described previously [29,30]. Briefly, mouse bone marrow cells isolated from the femur and tibia of 6- to 8-week-old C57BL/6 mice were cultured in α-MEM containing 10% FBS and M-CSF (30 ng/mL). After three days, the adherent BMMs were used as osteoclast precursors. BMMs were incubated with M-CSF (10 ng/mL) and RANKL (20 ng/mL) in the presence or absence of PF-3845 (1–10 μM) for four days to examine the effect on osteoclast differentiation. The cells were then fixed in 4% paraformaldehyde and stained using an Acid Phosphatase, Leukocyte (TRAP) staining kit (Sigma-Aldrich, St. Louis, MO, USA) for the quantitative evaluation of osteoclast formation. TRAP-positive MNCs containing more than three nuclei were scored as osteoclasts.

### 4.3. Quantitative Real-Time PCR Analysis

RNA was isolated using TRI solution (Bio Science Technology, Daegu, Korea), and 1 μg of the isolated RNA was reverse transcribed using SuperScript II Reverse Transcriptase (Invitrogen, Carlsbad, CA, USA) to generate complementary DNA (cDNA). Real-time PCR was performed using a LightCycler 1.5 real-time PCR system (Roche Diagnostics, Basel, Switzerland) with SYBR Premix Ex Taq (Takara Bio Inc., Shiga, Japan). The primers used in this study were as follows: *TRAP (Acp5)*, 5′-TCCCCAATGCCCCATTC-3′ and 5′-CGGTTCTGGCGATCTCTTTG-3′; *Ctsk*, 5′-GGCTGTGGAGGCGGCTAT-3′ and 5′-AGAGTCAATGCCTCCGTTCTG-3′; *Dcstamp*, 5′-CTTCCGTGGGCCAGAAGTT-3′ and 5′-AGGCCAGTGCTGACTAGGATGA-3′; *Nfatc1*, 5′-ACCACCTTTCCGCAACCA-3′ and 5′-TTCCGTTTCCCGTTGCA-3′.

### 4.4. Immunoblot Analysis

Cells were lysed in lysis buffer (iNtRON Biotechnology, Seongnam, Korea) containing protease and phosphatase inhibitors (GenDEPOT, Barker, TX, USA). After measuring protein concentrations with a BCA protein assay kit (Pierce Biotechnology, Rockford, IL, USA), equal amounts of protein were loaded onto 10% sodium dodecyl sulfate-polyacrylamide gels for electrophoresis, and the separated proteins were transferred to nitrocellulose membranes (Whatman, Florham Park, NJ, USA). The membranes were immersed in 3% non-fat dry milk in TBS-T (25 mM Tris-HCl (pH 7.4), 150 mM NaCl, and 0.1% Tween 20) to prevent non-specific binding of antibodies, and then incubated with primary antibodies at 4 °C. The next day, the membranes were probed with secondary antibodies, followed by chemiluminescent (ECL) visualization. Antibodies against phospho-p38, p38, phospho-MEK, phospho-ERK, ERK, phospho-JNK, phospho-IκBα, and phospho-c-RAF were obtained from Cell Signaling Technology (Danvers, MA, USA). Antibodies for NFATc1, cathepsin K, and β-actin were purchased from Santa Cruz Biotechnology (Santa Cruz, CA, USA), Millipore (Billerica, MA, USA), and Sigma-Aldrich (St. Louis, MO, USA), respectively.

### 4.5. Resorption Pit Assay

The resorption pit assay was performed as previously described [22,31]. Briefly, BMMs were plated on bone slices (IDS Nordic, Herlev, Denmark) and cultured in osteoclast induction medium for three days. Then, the cells were treated with PF-3845 (10 μM) or the vehicle for 24 h. Cells grown on bone slices were removed, and the slices were stained with Mayer’s hematoxylin to visualize the resorption pits. The area of the resorption pits was quantified using the i-Solution image analysis software (IMT i-Solution, Daejeon, Korea).

### 4.6. Immunofluorescence

BMMs plated on glass coverslips were cultured in osteoclast induction medium containing M-CSF (10 ng/mL) and RANKL (20 ng/mL) with PF-3845 (10 μM) or the vehicle. After four days, the cells were fixed and incubated with an anti-NFATc1 monoclonal antibody. NFATc1 signals were detected using an Alexa Fluor-488-conjugated secondary antibody. After washing, cell nuclei were stained with 4′,6-diamidino-2-phenylindole dihydrochloride (DAPI; Santa Cruz Biotechnology, Santa Cruz, CA, USA), and F-actin was visualized using rhodamine-conjugated phalloidin (Cytoskeleton, Denver, CO, USA). The cells were visualized with a Leica DM 2500 fluorescence microscope (Leica Microsystems, Wetzlar, Germany).

### 4.7. Ligature-Induced Experimental Periodontitis

Twenty male C57BL/6 mice were divided into four groups: nonligation with vehicle (NL + V), nonligation with PF-3845 (NL + PF-3845), ligation with vehicle (L + V), and ligation with PF-3845 (L + PF-3845). The mice in the ligation groups were tied with 5-0 silk ligatures around the maxillary second molars under anesthesia, as described previously, to induce alveolar bone destruction [21,22]. The vehicle or PF-3845 (10 mg/kg) was administered via intraperitoneal injection for six days. The treatment dose and administration route were selected using data obtained from previous research [8,32,33]. No mice exhibited abnormal behavior, sickness, or distress. The maxillae were collected after the mice were sacrificed on day seven, and the isolated maxillae were fixed in 4% paraformaldehyde.

### 4.8. Micro-Computed Tomography (Micro-CT) and Histomorphometric Analysis

The fixed upper jaws were scanned using a SkyScan 1272 high-resolution micro-CT system (Bruker, Kontich, Belgium), and the scanning parameters were set at 70 kV/142 µA with a resolution of 6 µm. Reconstructed 3D images were produced using CTvox software, and the distances between the CEJ and the ABC of the second molar (three sites: mesial, central, and distal sites) and bone volume fraction (bone volume/total volume, BV/TV) of the alveolar bone between the first and second molars were evaluated to assess the alveolar bone loss. The region of interest of volumetric analysis was determined using the roof of the furcation and the root apex as landmarks, as previously described [34]. After decalcification in 10% EDTA, the jaw samples were embedded in paraffin, and the sections (6 μm thickness) were stained with H&E and TRAP. The number of osteoclasts per bone perimeter was calculated.

### 4.9. Statistical Analysis

All experiments were performed in triplicate, and the data are presented as the mean ± standard deviation (SD). The two-tailed Student’s *t*-test or one-way analysis of variance with Tukey’s multiple comparison post hoc test were used to determine statistical differences (* *p* < 0.05, ** *p* < 0.01).

## Data Availability

Data can be obtained from the corresponding author.
